# The US Stock Market Leads the Federal Funds Rate and Treasury Bond Yields

**DOI:** 10.1371/journal.pone.0022794

**Published:** 2011-08-10

**Authors:** Kun Guo, Wei-Xing Zhou, Si-Wei Cheng, Didier Sornette

**Affiliations:** 1 Research Center on Fictitious Economy and Data Science, Chinese Academy of Sciences, Beijing, China; 2 School of Business, East China University of Science and Technology, Shanghai, China; 3 School of Science, East China University of Science and Technology, Shanghai, China; 4 Research Center for Econophysics, East China University of Science and Technology, Shanghai, China; 5 Department of Management, Technology and Economics, ETH Zurich, Zurich, Switzerland; 6 Swiss Finance Institute, c/o University of Geneva, Geneva, Switzerland; University of Minnesota, United States of America

## Abstract

Using a recently introduced method to quantify the time-varying lead-lag dependencies between pairs of economic time series (the thermal optimal path method), we test two fundamental tenets of the theory of fixed income: (i) the stock market variations and the yield changes should be anti-correlated; (ii) the change in central bank rates, as a proxy of the monetary policy of the central bank, should be a predictor of the future stock market direction. Using both monthly and weekly data, we found very similar lead-lag dependence between the S&P 500 stock market index and the yields of bonds inside two groups: bond yields of short-term maturities (Federal funds rate (FFR), 3M, 6M, 1Y, 2Y, and 3Y) and bond yields of long-term maturities (5Y, 7Y, 10Y, and 20Y). In all cases, we observe the opposite of (i) and (ii). First, the stock market and yields move in the same direction. Second, the stock market leads the yields, including especially the FFR. Moreover, we find that the short-term yields in the first group lead the long-term yields in the second group before the financial crisis that started in mid-2007 and the inverse relationship holds afterwards. These results suggest that the Federal Reserve is increasingly mindful of the stock market behavior, seen as key to the recovery and health of the economy. Long-term investors seem also to have been more reactive and mindful of the signals provided by the financial stock markets than the Federal Reserve itself after the start of the financial crisis. The lead of the S&P 500 stock market index over the bond yields of all maturities is confirmed by the traditional lagged cross-correlation analysis.

## Introduction

Financial markets play a more and more important role in the economic system. Many financial variables have predictive power for output or inflation of the real economy. Financial markets are becoming increasingly important to the real economy due to their impact on output growth and inflation, among others [1]–[6]. As an important part of financial markets, stock markets can be considered as economy barometers [7], [8]. As a consequence, monetary policy, which is usually based on inflation target and sometimes unemployment goals, is not independent of stock markets. There is a large number of financial economic literature concerned with the impact of and relationship between the monetary policy of central banks and the performance of stock markets. The common wisdom asserts that (i) the stock market variations and bond yield changes should be anti-correlated and (ii) the change in short-term interest rates, as a proxy of the monetary policy of the central bank, should be a predictor of the future stock market direction. The first assertion reflects the impact of capital cost on economic growth. The second statement is a corollary of the causal effect of the former one.

Some of the most relevant results for our study that were obtained by previous scholars on these two statements include the following. Tobin's portfolio selection theory [9] explained the stock price increases observed in times when the interest rate goes down as due to investors' preference for the higher yield of stock markets. Rigobon and Sack [10] documented that an increase in short-term interest rate results in a decline in stock prices and in an upward shift and flatter yield curve. Bernanke and Kuttner [11] found that a hypothetical unanticipated 25-basis-point cut in the FFR target is associated with approximately a 1% increase in the broad stock indexes. Bjørnland and Laitemo [12] found a significant relationship, which is however the inverse of (i) and (ii): a one percent increase of the stock market leads on average to a 4-basis-point increase of the interest rate. Two of us have also previously found that the stock market seems to influence the FFR, during the 2000–2003 US stock market antibubble [13]–[16].

Here, using an extension of the so-called TOP technique [13]–[16] for the joint analysis of pairs of time series, we revisit the pertinence of these two assertions (i) and (ii) by estimating the lead-lag structure between the US stock market proxied by the S&P 500 index and a set of Treasury bond yields, including the Federal funds rate (FFR), which constitutes one of the tools implementing monetary policy in the US. Our analysis is applied to monthly and weekly data of Federal funds effective rate (FFR), and nine Treasury bond yields with different maturities: 3M (3 months), 6M, 1Y (1 year), 2Y, 3Y, 5Y, 7Y, 10Y, and 20Y. The period of analysis from August 2000 to February 2010 includes the bearish market up to mid-2003, the bullish bubble-like market regime up to October 2007 followed by the turbulent phases associated with the so-called great Recession [17], [18]. Given the extraordinary developments associated with the financial crises followed by economic crises in different parts of the world, it is particularly interesting to investigate the lead-lag structure between the US stock market and a set of Treasury bond yields.

## Materials and Methods

### Description of the thermal optimal path (TOP) method

The thermal optimal path (TOP) method has been proposed as a new method to identify and quantify the time-varying lead-lag structure between two time series. The TOP method was successfully applied to several economic cases [14]–[16]. It works as follows.

Consider two standardized time series 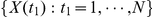 and 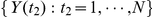. The matrix 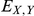 of distances between 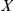 and 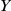 is defined as [14], [15]

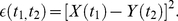
(1)The element 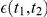 of the matrix 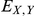 thus compares the realization 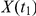 of 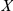 at time 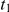 with the realization 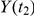 of 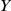 at time 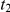. The value 
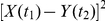
 defines the distance between the realizations of the first time series at time 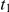 and the second time series at time 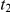. The 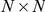 matrix 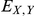 thus embodies all possible point-wise pairwise comparisons between the two time series. Note that the distance matrix could be modified to deal with two non-monotonic time series, for which the TOP algorithm is essentially the same [16].

**Figure 1:**
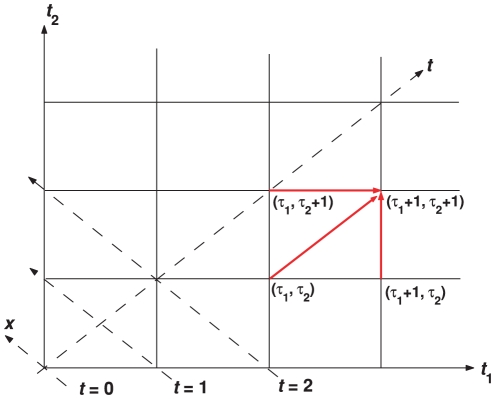
Thermal optimal path method. Representation of the two-layer approach in the lattice 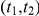 and of the rotated frame 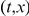 as defined in the text. The three arrows depict the three moves that are allowed to reach any node in one step.

Once the matrix 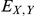 with elements given by Eq. (1) is obtained, an optimal path is determined that quantifies the lead-lag dependence between the two time series. Figure 1 gives a schematic representation of how lead-lag paths are defined [14]. The first (resp. second) time series is indexed by the time 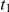 (resp. 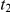). The nodes of the plane carry the values of the distance for each pair 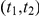. The path along the diagonal corresponds to taking 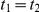, i.e., compares the two time series at the same time. Paths above (resp. below) the diagonal correspond to the second time series lagging behind (resp. leading) the first time series. The figure shows three arrows which define the three causal steps (time flows from the past to the future both for 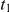 and 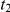) allowed in our construction of the lead-lag paths. A given path selects a contiguous set of nodes from the lower left to the upper right. The relevance or quality of a given path with respect to the detection of the lead-lag relationship between the two time series is quantified by the sum of the distances along its length, called the “cost” of the path. The lead-lag structure is then obtained as the relationship 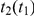 as a function of 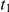, as described shortly. We stress that the two-layer scheme presented in Fig. 1 performs better than multi-layer schemes [15].

As shown in Fig. 1, it is convenient to use the rotated coordinate system 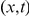 such that
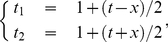
(2)where 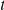 is in the main diagonal direction of the 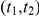 system and 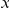 is perpendicular to 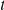. The origin 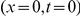 corresponds to 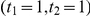. Then, the standard reference path is the diagonal of equation 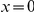, and paths which have 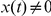 define varying lead-lag patterns. Inverting (2), we have
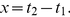
(3)This means that a positive 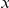 corresponds to 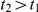, which by definition of the optimal thermal path below means that the second time series 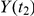 lags behind the first time series 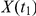, or equivalently 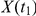 leads 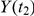.

The idea of the TOP method is to identify the lead-lag relationship between two time series as the best path in a certain sense. A natural idea is that the best path is the one which has the minimum sum of its distances along its length (paths are constructed with equal lengths so as to be comparable). This path with minimum cost has thus the minimum average distance between the two time series, i.e., it is such that 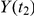 resembles the most 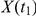 along this path 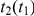. The problem with this idea is that the noises decorating the two time series introduce spurious patterns which may control the determination of the path which minimizes the sum of distances, leading to incorrect inferred lead-lag relationships. It has been shown that a robust lead-lag path is obtained by defining an average over many paths, each weighted according to a Boltzmann-Gibbs factor, hence the name “thermal” optimal path method [14]–[16]. Intuitively, this corresponds to performing an averaging operation over neighboring paths of almost the same cost.

Concretely, we first calculate the partition functions 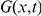, for all values of 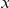 at a fixed 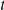 in the lattice shown in Fig. 1, and their sum 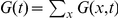 so that 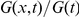 can be interpreted as the probability for a path to be at distance 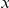 from the diagonal for a distance 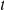 along the diagonal. This probability 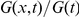 is determined as a compromise between minimizing the mismatch or cost as defined above (similar to an “energy”) and maximizing the combinatorial weight of the number of paths with similar mismatches in a neighborhood (similar to an “entropy”). As illustrated in Fig. 1, in order to arrive at 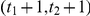, a path can come from 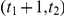 vertically, 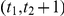 horizontally, or 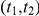 diagonally. The recursive equation on 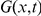 is therefore
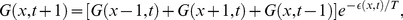
(4)where 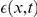 is defined by Eq. (1). The parameter 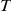 plays the role of a “temperature” controlling the relative importance of cost versus combinatorial entropy. The larger 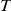 is, the larger the number of paths that contribute to the partition functions. In contrast, as 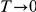, only the path with minimal cost counts. The recursion relation (4) is derived following the work of Wang et al. [19]. To get 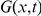 at the 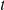-th layer, we need to know and bookkeep the previous two layers from 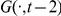 to 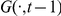. After 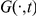 is determined, these values are normalized by 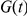 so that 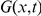 does not diverge at large 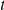. The boundary condition of 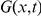 plays an crucial role. For 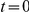 and 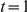, 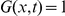. For 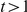, the boundary condition is taken to be 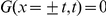, in order to prevent paths to remain on the boundaries.

Once the partition functions 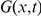
's have been calculated, we can obtain any statistical average related to the positions of the paths weighted by the set of 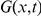
's. For instance, the local time lag 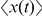 at time 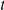 is given by
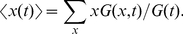
(5)Expression (5) defines 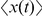 as the thermal average of the local time lag at 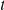 over all possible lead-lag configurations suitably weighted according to the exponential of minus the measure 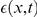 of the similarities of two time series. For a given 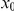 and temperature 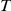, we determine the thermal optimal path 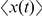. We can also define an “energy” or cost 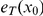 to this path, defined as the thermal average of the measure 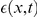 of the similarities of two time series:
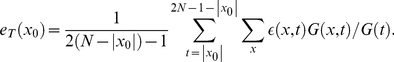
(6)


### Bootstrapping tests and statistical significance

In order to test whether the extracted lead-lag structure is statistically significant, we introduce a bootstrap approach [20] that is specifically adapted to the present problem. This statistical test extends and makes more robust the method and results, as compared with previous works [14], [15], [16]. Consider two time series 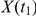 (for instance the logarithmic returns of S&P 500) and 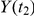 (for instance the time increments of bond yields). We perform the TOP analysis on a fixed time interval at some temperature 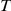. Let us assume we obtain the lead-lag function 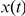. Recall that 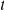 is the diagonal of the 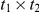 plane. We then shuffle 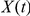 and 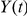 and redo the TOP analysis at the same temperature 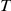. We obtain a new lead-lag function 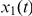. This process is repeated another 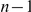 times, giving a total of 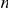 paths 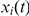 with 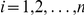. A typical value of 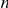 used below is 1000. For each 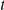, out of the 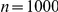 reshuffled time series, we determine the 5% quantile 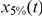 and the 95% quantile 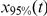, denoted in the following as 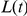 and 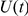. If 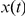 is smaller than 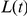 or larger than 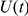, we interpret that the lead-lag 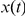 at time 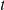 is different from zero at the significance level of 95% or larger. Complementarily, given the obtained lead-lag 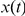, out of the 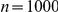 reshuffled time series, we obtain the 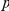-value as a function of 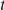, which thus characterizes the time periods when there is a statistically significant lead-lag structure as those with small 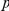-values.

### Data sets

In the following, we apply the TOP method respectively to monthly and weekly data of the S&P 500 index, Federal funds effective rate (FFR), and nine Treasury bond yields with different maturities: 3M (3 months), 6M, 1Y (1 year), 2Y, 3Y, 5Y, 7Y, 10Y, and 20Y. Each time series spans from August 2000 to February 2010. The Treasury bond at 30-year maturity is not considered because it was discontinued in January 2002 and then reintroduced in February 2006.

**Figure 2:**
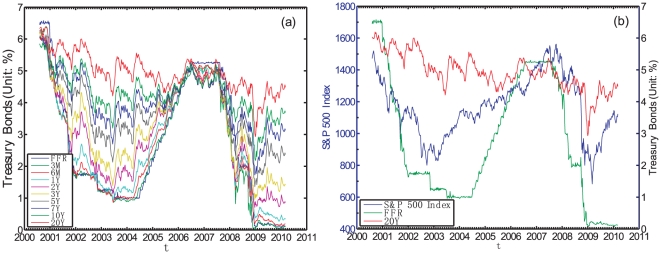
Data sets. (a) Weekly sampling of the Federal effective funds rate (FFR) and nine Treasury bond yields. (b) S&P 500 and FFR together with the 20Y for comparison.

Figure 2 shows the weekly sampling of the FFR, the nine Treasury bond yields with different maturities, and the S&P 500 index. In the left panel of Fig. 2, very interesting patterns emerge in the term structure. In general, the yields of Treasury bonds with short maturities are more sensitive to the economic circumstance and change to a larger extent. In 2000, 2006 and 2007, the spread is very narrow and the FFR is even higher than the Treasury bond yields, corresponding to an anomalous inverted yield curve. These time periods correspond respectively to the early stages of the 2000 US stock market crash and to the current financial crisis. The spread reaches local maxima in 2004 and 2010. In addition, the right panel of Fig. 2 suggests that the FFR and the S&P 500 index change roughly in the same direction. It is thus interesting to refine this visual impression and determine rigorously using the TOP method described above what is the lead-lag structure between the evolution of the US stock market and the FFR, which embodies an important part of the policy of the Federal Reserve.

In this paper, we use as inputs the logarithmic returns of the S&P 500 index and the increments of the FFR and of all the yields, rather than the non-stationary original time series. We define the logarithmic returns of the S&P 500 index as follows
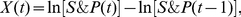
(7)and the logarithmic increments of yields curves as follows
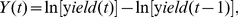
(8)where the time unit for 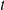 is one week for weekly data and one month for monthly data. We then normalize the two time series 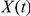 and 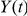 so that their mean is zero and their standard deviation is equal to 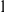 [14]. This ensures that they are comparable and can be used meaningfully in the TOP analysis to extract their lead-lag structure.

### Unit root tests

We perform unit root tests on the logarithms of the original time series and their first-order differences (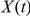 and 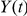) to check for their stationary. The augmented Dickey-Fuller (ADF) [21], Phillips-Perron (PP) [22], and Kwiatkowski-Phillips-Schmidt-Shin (KPSS) [23] tests are adopted. For the ADF and PP tests, the null hypothesis is that the time series has a unit root, which utilizes the 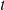-statistic. In contrast, the null hypothesis of the KPSS method is that the time series is stationary and uses the LM-statistic. The results are presented in Table 1.

**Table 1:** Unit root tests of the logarithmic monthly and weekly data and their first-order differences.

	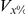			S&P 500		FFR		1Y		5Y		20Y	
method	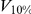	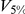	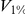	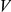	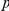	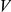	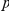	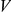	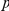	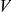	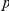	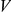	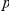
Logarithmic monthly data													
ADF	−2.58	−2.89	−3.49	−2.14	0.23	−0.56	0.87	−0.64	0.86	−2.27	0.16	−2.51	0.12
PP	−2.58	−2.89	−3.49	−2.15	0.23	−0.01	0.95	−0.23	0.93	−2.00	0.27	−2.51	0.12
KPSS	0.35	0.46	0.74	0.16	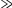 0.1	0.31	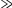 0.1	0.27	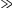 0.1	0.43	0.06	1.02	0.00
Logarithmic weekly data													
ADF	−2.57	−2.86	−3.44	−2.08	0.25	−0.16	0.94	−0.29	0.92	−2.10	0.21	−2.95	0.04
PP	−2.57	−2.86	−3.44	−2.08	0.25	−0.00	0.96	−0.30	0.92	−2.04	0.27	−2.72	0.07
KPSS	0.35	0.46	0.74	0.31	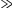 0.1	0.69	0.01	0.62	0.02	0.84	0.00	1.91	0.00
Difference of logarithmic monthly data													
ADF	−2.58	−2.89	−3.49	−8.29	0.00	−4.21	0.00	−6.68	0.00	−8.07	0.00	−9.35	0.00
PP	−2.58	−2.89	−3.49	−8.35	0.00	−5.36	0.00	−6.66	0.00	−8.07	0.00	−9.83	0.00
KPSS	0.35	0.46	0.74	0.11	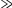 0.1	0.32	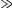 0.1	0.32	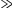 0.1	0.10	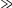 0.1	0.05	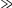 0.1
Difference of logarithmic weekly data													
ADF	−2.57	−2.86	−3.44	−22.5	0.00	−24.6	0.00	−16.6	0.00	−19.3	0.00	−17.4	0.00
PP	−2.57	−2.86	−3.44	−22.5	0.00	−24.6	0.00	−16.8	0.00	−19.4	0.00	−17.4	0.00
KPSS	0.35	0.46	0.74	0.13	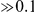	0.35	0.10	0.31	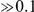	0.07	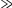 0.1	0.04	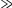 0.1

The augmented Dickey-Fuller (ADF), Phillips-Perron (PP) and Kwiatkowski-Phillips-Schmidt-Shin (KPSS) tests are adopted. 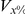 is the critical value at the 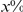 significance level, 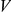 is the statistic, and 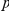 is the 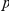-value.

For the logarithmic monthly data and logarithmic weekly data, the ADF and PP tests show that these time series are not stationary and have a unit root since the 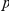-values are greater than 10%, except for the 20Y yield. In contrast, the KPSS test suggests that four time series are stationary since the 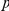-values are much greater than 10%.

For the differences of the logarithmic monthly data and logarithmic weekly data, the ADF and PP tests show that all time series are stationary at the 1% significance level, and the KPSS test also confirms that these time series are stationary at the 10% level. These results justify our use of the logarithmic returns in the TOP analysis in order to avoid possible spurious signals in the estimated lead-lag structure that could result from large excursions exhibited by the non-stationary time series.

## Results

### The S&P500 leads all yields: Evidence from the TOP method

#### Empirical results

**Figure 3:**
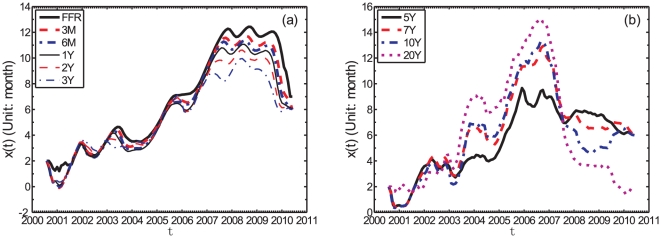
Lead-lag 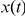 for monthly data. Dependence of the lead-lag 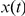 between the returns of the S&P500 index taken as the first time series and the logarithmic variation of each of the yields for the monthly data: (a) FFR, 3M, 6M, 1Y, 2Y, and 3Y Treasury bond yields as the first group; (b) 5Y, 7Y, 10Y, and 20Y bond yields as the second group. The unit of 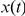 is one month.

Figure 3 shows the instantaneous evolution of the lead-lag 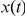 between the returns of the S&P500 index taken as the first time series and the logarithmic variation of each of the yields for the monthly data at temperature 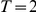. We have been careful to investigate the impact of the locations of the starting and ending extremities of the paths. There are indeed a total of 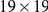 thermal optimal paths, because there are 19 starting points 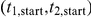 and 19 ending points 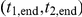, denoted using the 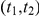 system instead of the 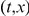 system for simplicity. The 19 starting points are 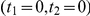, 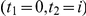, and 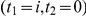 for 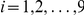. The 19 ending points are 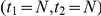, 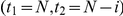 and 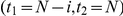 for 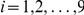, where 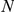 is the length of the time series. The overall thermal optimal path 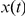 is chosen as the one with minimal energy (or total cost) among the 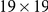 thermal paths. As for the choice of the temperature 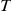, we investigated other values and found our results to be robust and qualitatively similar with respect to variations of 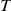 between 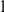 and 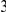. To present our results, we choose this value 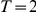 as it seems to represent a reasonable optimal, confirmed by cross-correlation analyses performed on the steady periods found with fixed lag times for various 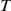
's.

Figure 3 is organized in two panels, each panel plotting one group. The first group includes FFR, 3M, 6M, 1Y, 2Y, and 3Y Treasury bonds as shown in Fig. 3(a). The second group includes 5Y, 7Y, 10Y, and 20Y Treasury bonds as shown in Fig. 3(b). The evolution of 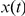 in each group are quantitatively similar.

**Figure 4:**
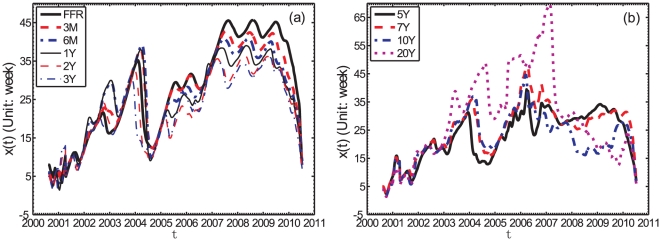
Lead-lag 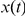 for weekly data. Dependence of the lead-lag 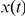 between the returns of the S&P500 index taken as the first time series and the logarithmic variation of each of the yields for the weekly data: (a) FFR, 3M, 6M, 1Y, 2Y, and 3Y Treasury bond yields as the first group; (b) 5Y, 7Y, 10Y, and 20Y bond yields as the second group. The unit of 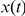 is one week.

Figure 4 is the same as Fig. 3 for weekly data. Apart from largest fluctuations of the lead functions, the results are very similar and robust to this change of time scale from monthly to weekly.

#### Statistical significance

**Figure 5:**
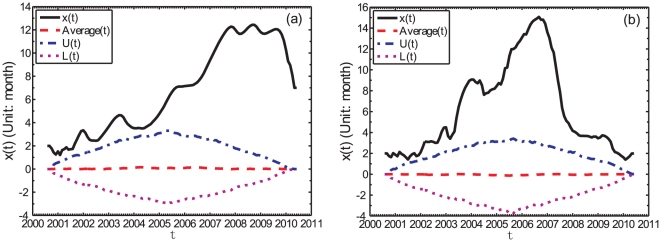
Bootstrap test for the significance of the lead-lag structure. (a) the monthly FFR and (b) the monthly 20Y Treasury bond yield.

Before commenting and exploiting the information presented in Figs. 3 and 4, it is important to ascertain their statistical significance. For this, we use the bootstrap method described above. Figure 5 illustrates the obtained results from the monthly data for two maturities, namely the shortest one (FFR) and the longest one (20-year Treasury bond yield). It shows that the two lead function 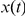 are well above the 95% quantile curves, that is, 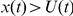. The conclusion is the same for other Treasury bond yields. We conclude that the obtained lead-lag structure for the monthly data cannot be produced by chance at the 95% significance level.

**Figure 6:**
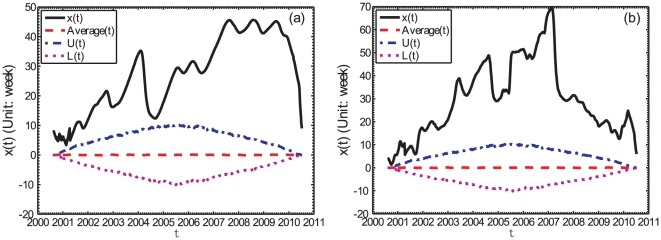
Bootstrap test for the significance of the lead-lag structure. (a) the weekly FFR and (b) the weekly 20Y Treasury bond yield.

Figure 6 illustrates the obtained results from the weekly data for two maturities, namely the shortest one (FFR) and the longest one (20-year Treasury bond yield). The conclusion is the same for other Treasury bond yields. Therefore, the 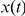 functions for the weekly data are positive at the 95% significance level, which unveils the nontrivial intrinsic lead-lag structure of the S&P 500 index and the yield time series.

#### Two shocking stylized facts

The first and most important observation extracted from Figs 3 and 4 is that, for all yields and at all times, the S&P500 index leads the yield changes, since 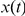 is always positive, which by definition (3), means that 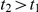 for the optimal thermal path. Since the index 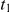 corresponds to the S&P500 index and the index 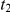 corresponds to one of the yields, this conclusion follows. This result confirms and extends considerably that reported previously by two of us [13] using standard measures of correlations over a restricted period from 2001 to 2003, under the somewhat provocative title “Causal slaving of the U.S. Treasury Bond Yield … by the Stock Market…” Indeed, as this title suggests, this result 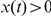 is particularly striking and rich of implication. This result collides against the common wisdom that usually asserts the following two rules:

- the stock market variations and the yield changes should be anti-correlated;
- the change in FFR, as a proxy of the monetary policy of the central bank, should be a predictor of the future stock market direction.

Indeed, according to the standard story, a lower interest rate means lower costs of borrowing for the private sector, implying that the private sector is going to profit from this opportunity by increased investments in innovations and entrepreneurial opportunities, leading (with some lag) to an improved outlook for the future growth of the economy. Since stock market prices reflect the anticipation of investors, this better outlook for the future economy should be soon reflected in the appreciation of the stock market. Reciprocally, an increase of the yields and in particular of the FFR should, according to the standard story, translate soon into a drag on the growth of stock markets.

We observe the opposite of (i) and (ii). First, we find that the stock market and yields move in the same direction, as pointed out independently by R. Werner [24]. Second, the stock market leads the yields, including and especially the FFR. The implication is clear: the central bank policy is (1) reacting to the stock market and (2) is following it. When the stock market exhibits a rally, the Fed tends to progressively increase its rates as an attempt to calm down the “overheating engine”, as occurred towards the end of the ICT bubble when the Fed rate was increased to 6.5%. A similar increase of the Fed rate occurred from 2004 to 2007. When the stock market plunges, the Fed tends to decrease its rates, in the hope of putting a brake on the stock market losses that negatively feedback onto the real economy via the wealth effect.

Both previous and present Fed chairmen Greenspan and Bernanke have increasingly made clear that the Federal Reserve does care more and more about the evolution of the stock markets. On Dec. 3rd, 2010, former Federal Reserve Chairman Alan Greenspan told CNBC that rising stock values have played a critical role in the economic recovery. The stock market got a boost from the Fed policy to boost liquidity, which drove interest rates down and pushed investors toward riskier investments like stocks. “I think we are underestimating and continuing to underestimate how important asset prices, very specifically equity prices, are not only to shareholders but the economy as a whole,” he said. Equities have risen more than 80% from the lows set during the financial crisis, noted Greenspan, benefiting investors and helping fuel the recovery (Source: http://www.dailyfinance.com/story/investing/greenspan-rising-stock-markets-are-key-to-recovery/19743325/?icid=sphere_copyright). On Nov. 3rd., 2010, Bernanke issued the following statement in an opinion article for the Washington Post released hours after the Fed announced the $600 billion of Treasury buying through June in a second round of unconventional monetary stimulus: “Resuming large-scale asset purchases should boost economic growth through lower borrowing costs and higher stock prices… Stock prices rose and long-term interest rates fell when investors began to anticipate this additional action… Easier financial conditions will promote economic growth.” Being content to see the stock market growing, this suggests a hidden mandate of the Federal Reserve to steer the stock markets.

It seems that the dynamics of the Fed policy, as translated in the Fed rates and the longer maturity yields (which of course are far from being controlled by the central bank), is much more straightforward than articulated in fancy models [25]. The evidence presented here suggests that Fed policy appears to be as if a straightforward reaction to financial markets was the main factor.

#### Comparison between different yields

Comparing the lead functions 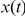 for the various yields with different maturities, we find that the short-term yields in the first group (left panel of Figs. 3 and 4) move approximately in synchrony with the long-term yields in the second group (right panel of Figs. 3 and 4) until 2007. And this synchrony is almost perfect from the yields spanning FFR to 3Y in the first group until mid-2007. Thereafter, during the time period following the financial crisis that started in mid-2007, we can observe that the short-term yields clearly lead the long-term yields and we have the sequence of inequalities
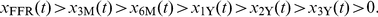
(9)This is seen from the fact that 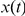 tends to be larger for the short-term yields, since they are all compared with the same S&P500 stock market index. It is also interesting to observe the increasing lag 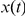 between the yield rates and the S&P500 index from around 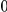 month in 2000 to about one year in 2007. This is followed by a plateau for all yields from FFR to 3Y, that lasts about 2 years and is then followed by a decay of the lag thereafter to about half its maximum, i.e., around 6 months.

For the second group of yields with maturities from 5Y to 20Y whose 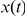
's are plotted in the right panel of Figs. 3 and 4, the picture is somewhat different. Before early 2003, the four curves are close to each other with no clear lead-lag structure between them. Then, from 2003 to mid-2007, a period corresponding to a very bullish upward trend of the stock market boosted by the favorable low rate of the Fed policy and a booming real-estate bubble, one can observe that the longer term yields lead clearly the shorter term yields:
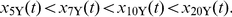
(10)Thereafter, in the reaction to the financial crisis, one observes as for the FFR-3Y yields that the shorter-term yields lead the long-term yields:
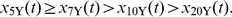
(11)There is much less evidence for a plateau of the lead structure with respect to the S&P500.

We would also like to mention that a reversal such as the one from (10) to (11) does not seem to have been documented before.

### The S&P500 leads all yields: Evidence from cross-correlation analysis

By construction, the traditional cross-correlation analysis [26] is not adapted to time-varying lead-lag structures. It is however useful to investigate how it performs in the present context in which the TOP method has diagnosed a significant time-varying structure. For centered random variables, the cross-correlation function can be calculated as follows:
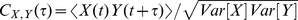
(12)where 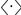 denotes the sample average and 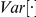 is the sample variance.

**Figure 7:**
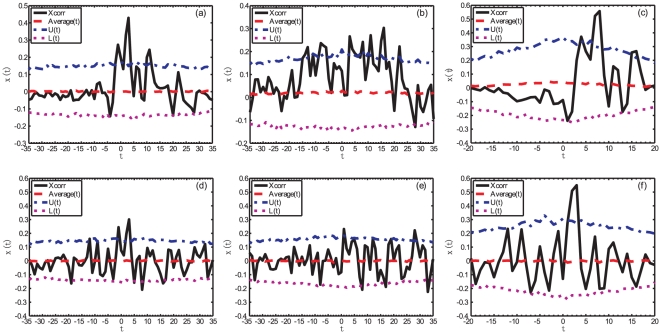
Lagged cross-correlation analysis. Lagged cross-correlation between the logarithmic return of the S&P 500 index and the logarithmic difference of the FFR (a–c) and between the logarithmic return of the S&P 500 index and the logarithmic difference of the 20Y Treasury bond yield (d–f) during different time periods: (a,d) the whole time period from August 2000 to February 2010, (b,e) the time interval from August 2000 to April 2007, and (c,f) the time interval from May 2007 to February 2010. The ordinate axis shows the cross-correlation coefficients 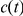. The unit of the lag time 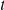 along the abscissa is month.

Two representative time series (FFR and 20Y) are presented for illustration. The significance levels of the cross-correlations are evaluated using bootstrapping tests through shuffling the return time series, similar to the analysis for the TOP method. We use the monthly data in this analysis. For each pair of time series, we analyze the whole time series and two non-overlapping time periods. The results are shown in Fig. 7. It is obvious that the lagged cross-correlation analysis is not able to characterize the instantaneous evolution of the lead-lag structure evidenced in the previous TOP analysis. This is not a surprise.

#### The (S&P 500, FFR) pair

For the (S&P 500, FFR) pair in the whole time period, the highest peak found in Fig. 7(a), with a positive lag, shows that the FFR lags behind the S&P 500 index by about 3 months, with a cross-correlation coefficient 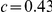, which is significantly positive at the confidence level of 95%. There are two other peaks that are also significant, one at a negative lag of 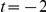 month with 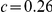 and another at the positive lag 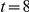 month with 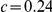. The largest peak with positive lag and highest cross-correlation coefficient 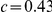 can be considered as confirming the main results of the previous section that the stock market changes precede the FFR variations. Due to the fixed lead-lag structure of the method, the cross-correlation provides only an average coarse representation of the real much richer and dynamical nature of the lead-lag structure.

For the time period before April 2007, we see many peaks at positive and negative lags 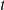 that are significantly different from zero, as shown in Fig. 7(b). It is hard to extract from this plot a clear picture about the lead-lag structure between the S&P 500 and FFR. In the presence of large variations of the lead-lag structure, it is not surprising that the cross-correlation analysis is not informative.

For the time period after April 2007, we see in Fig. 7(c) a significant peak at the positive lag 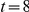 month with 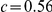. This lag is consistent in magnitude with the average value of the 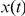 curve shown in Fig. 5(a). This clear signal in the cross-correlation analysis can be explained from the fact that the lead-lag has stabilized approximately above a value of 6 months, according to the analysis of the 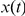 function shown in Fig. 5(a) during the time period under investigation.

#### The (S&P 500, 20Y) pair

For the (S&P 500, 20Y) pair in the whole time period, there are two significant peaks around zero lag 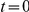 in Fig. 7(d).

For the time period before April 2007, the signal is ambiguous although we can see several significant peaks in Fig. 7(e).

For the time period after April 2007, we see in Fig. 7(f) only one significant peak at 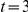 month with 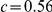. According to Fig. 5(b), the lead-lag 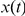 decreases from about 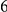 to 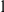 month. Therefore, these two analyses give consistent results: on average, the S&P 500 index leads the 20Y Treasury bond yield by about 3 months.

Comparing Fig. 7 for the cross-correlation analysis and Fig. 5 for the TOP analysis, we can conclude that the cross-correlation analysis can extract only part of the information and the TOP method is clearly superior.

## Discussion

In this work, we have adopted the thermal optimal path method to investigate the dynamics lead-lag structure between the S&P 500 index of the US stock market and Federal Funds rate, as well as several Treasury bond yields with different maturities. The time period that has been investigated runs from August 2000 to February 2010. Both monthly and weekly data have been used and we obtained consistent results. In all cases, the S&P 500 index is found to lead the FFR and the bond yields. This is quantified by the lead function 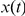 found to be positive at a high statistical confidence level determined by bootstrapping tests. This finding is consistent with and extends significantly a previous work reporting that the US Federal Reserve was “slaved” to the stock market during the 2000–2003 US stock market antibubble [13].

According to the TOP analysis, we observed that the FFR and the Treasury bond yields can be divided into two groups. The first group contains FFR, 3M, 6M, 1Y, 2Y, and 3Y bond yields with short-term maturities and the second group contains 5Y, 7Y, 10Y, and 20Y bond yields with long-term maturities. The lead functions 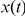 between the S&P 500 index and the yields in each group have very similar quantitative shapes, while they are different at a quantitative level across the two groups. We found that the short-term yields in the first group lead the long-term yields in the second group before the current financial crisis around 2007 and the inverse relationship holds afterwards, namely the long-term yields lead the short-term yields after 2007.

For the first group, the lead function 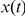 increases during the time period from 2000 to 2007, followed by a two-year-long plateau, and then plummets in late 2009. We also found that the yields (including FFR) with shorter maturity in the first group have a longer lag behind the S&P 500 index than for the longer maturities. In contrast, for the second group, the lead function 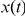 increases till 2006 and then decreases. We observed a reversal of the order of the lead functions 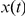 among the different maturities in 2007: a yield with shorter maturity has a shorter lag to the S&P 500 index before the reversal point and a yield with longer maturity has a shorter lag to the S&P 500 index after the reversal point. Qualitatively, the reversal phenomenon is coincident with the outbreak of the current financial crisis.

The lag of the FFR to the S&P 500 index can be interpreted in the light of comments of the previous and present Fed chairmen Greenspan and Bernanke that the growth of stock markets is “key” to the recovery and health of the economy. The evidence provided here suggests indeed that the FFR policy is in a significant part influenced by the recent past behavior of the stock markets (stock market 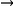 Federal Funds rate). In plain words, the fact that the FFR follows the stock market direction can be interpreted as a direct attempt to limit its losses and revive it in times of bearish markets or to stabilize it in times of overly buoyant bubbling markets.

As for the longer maturities, the lag structure with respect to the S&P 500 index reflects (i) a natural link in the term-structure that attach the longer maturities to the shortest maturity and (ii) the aggregate strategies of investors facing uncertainties over the long term behavior of the economy [27], [28]. In the first sub-stage before 04/2007, we observe the causal relational flow from the stock market 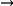 Federal Funds rate 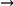 short-term yields 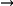 long-term yields, and afterwards, we find the flow from the stock market 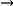 long-term yields 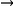 short-term yields 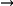 Federal funds rate. Thus, the lead-lag structures between the different yields changed after the financial crisis starting in 2007. This change can be rationalized by the strategies implemented by long-term investors in the face of growing global market uncertainties, such as central banks of major Asian countries and pension funds which are heavily invested in the US long-term Treasury bonds [29]. The stern challenges faced by the US economy escalated the uncertainty which cascaded to exchange rate and inflation. Consequently, the long-term Treasury bonds became quite reactive to the behavior of stock markets, reflecting the actions of these long-term investors “flying to safety”: a plunge in the stock markets led to strong demand for the supposedly safe US Treasury bonds, pushing down mechanically the corresponding yields. This suggests that the long-term investors have been more reactive and mindful of the signals provided by the financial stock markets than the Federal Reserve itself after the start of the financial crisis. This may be due to the more complex agenda as well as the delicate role of the Federal Reserve, which has to take into account the impact of its interventions [25]. Caution and prudence on the part of the Fed in a time of high uncertainty may thus be the reason for this inversion of the lead-lag relationship between changes of yields of different maturities. However, the robust lead of the S&P 500 stock market index with respect to yields of all maturities remains the most important stylized fact unearthed by our study.
